# Household Food Insecurity, Lung Function, and COPD in US Adults

**DOI:** 10.3390/nu13062098

**Published:** 2021-06-19

**Authors:** Francisca de Castro Mendes, Kirstie Ducharme-Smith, Gustavo Mora-Garcia, Saleh A. Alqahtani, Maria Stephany Ruiz-Diaz, Andre Moreira, Rodrigo Villegas, Vanessa Garcia-Larsen

**Affiliations:** 1 Serviço de Imunologia Básica e Clínica, Departamento de Patologia, Faculdade de Medicina, Universidade do Porto, 4200-319 Porto, Portugal; francisca_castromendes@hotmail.com (F.d.C.M.); andremoreira.fmup@gmail.com (A.M.); 2 EPI Unit, Instituto de Saúde Pública, Universidade do Porto, 4050-091 Porto, Portugal; 3 Program in Human Nutrition, Department of International Health, The Johns Hopkins Bloomberg School of Public Health, Baltimore, MD 21205, USA; kduchar1@jhmi.edu; 4 Department of Family Medicine and Public Health, Faculty of Medicine, Universidad de Cartagena, #24- a Carrera 50a #2463, Cartagena de Indias 130001, Colombia; gmorag@unicartagena.edu.co; 5 King Faisal Specialist Hospital & Research Centre, Riyadh 11564, Saudi Arabia; salqaht1@jhmi.edu; 6 Division of Gastroenterology and Hepatology, Department of Medicine, Johns Hopkins University, Baltimore, MD 21205, USA; 7 Center for Innovation and Research in Diabetes and Metabolism—INNOVATID, Calle 28 20 36, Cartagena de Indias 130001, Colombia; mruizd2@unicartagena.edu.co; 8 Serviço de Imunoalergologia, Centro Hospitalar São João, 4200-319 Porto, Portugal; 9 Faculdade de Ciências da Nutrição e Alimentação, Universidade do Porto, 4150-180 Porto, Portugal; 10 Biostatistics Unit, School of Public Health, University of Chile, Independencia Santiago 939, Chile; villegas.ro@gmail.com

**Keywords:** chronic obstructive pulmonary disease (COPD), diet, food insecurity, NHANES, lung function, adults

## Abstract

Increasing epidemiological evidence suggests that optimal diet quality helps to improve preservation of lung function and to reduce chronic obstructive pulmonary disease (COPD) risk, but no study has investigated the association of food insecurity (FI) and lung health in the general population. Using data from a representative sample of US adults who participated in the National Health and Nutrition Examination Survey (NHANES) 2007–2012 cycles, we investigated the association between FI with lung function and spirometrically defined COPD in 12,469 individuals aged ≥ 18 years of age. FI (high vs. low) was defined using the US Department of Agriculture’s Food Security Scale). Population-weighted adjusted regression models were used to investigate associations between FI, and forced expiratory volume in 1 s (FEV_1_), forced vital capacity (FVC), their ratio, and spirometrically defined restriction (FVC below the lower limit of normal) and airflow obstruction (COPD). The prevalence of household FI was 13.2%. High household FI was associated with lower FVC (adjusted β-coefficient −70.9 mL, 95% CI −116.6, −25.3), and with higher odds (OR) of spirometric restriction (1.02, 95% CI 1.00, 1.03). Stratified analyses showed similar effect sizes within specific ethnic groups. High FI was associated with worse lung health in a nationally representative sample of adults in the US.

## 1. Introduction

Poor lung function is the strongest predictor of all-cause mortality [1]. Chronic obstructive pulmonary disease (COPD) is the fourth leading cause of death worldwide [2], and its burden is greater in low- and middle-income countries (LMIC), where over 80% of mortality for this disease occurs [3]. Albeit tobacco smoking is the primary risk factor for poor lung function and COPD, current trends in smoking cessation do not align with the increasing burden of COPD, and increasing evidence supports a possible role of other modifiable environmental factors including pollution [4] and poverty [5].

Food insecurity (FI) is defined as “the inability to access sufficient safe and nutritious food in a socially acceptable manner, which is typically associated with precarious socioeconomic conditions” [6]. In developed countries, an estimated 8% to 20% of households are affected by FI [7]. The prevalence of FI is higher among ethnic minorities and low-income families, with these homes often relying on high energy-dense, and nutritionally poor foods (e.g., high in sugars, fats and sodium). These foods are often less expensive than higher quality nutritious foods [8], and these individuals often have a low consumption of fruits and vegetables [9]. 

Greater FI is associated with a higher prevalence and risk of several non-communicable diseases (NCDs), such as cardiometabolic and pulmonary diseases [10]. Increasing epidemiological evidence shows that a high-quality diet is associated with better lung function and with a lower risk COPD, although most of the evidence is from affluent populations [11]. Results from the Hertfordshire Cohort Study showed that a dietary pattern that included higher intake of fruit, vegetables, oily fish and whole grain cereals was associated with better lung function and lower prevalence of COPD [12]. Similarly, amongst US women [13] and men [14] a dietary pattern rich in fruit, vegetables, and fish was associated with a lower risk of newly diagnosed COPD. Observational evidence from population-based surveys in European adults have shown that a higher intake of fresh fruits, vegetables, and dietary sources of flavonoids has been prospectively associated with a slower decline in lung function [15], lower prevalence of COPD [16], and with better lung function [17].

To date, the relationship between FI and lung function has not been investigated in population-based studies in the US, a country where socio-economic disparities and food inequalities still represent a major public health challenge. Our study aimed to investigate the association of FI, lung function and COPD in adults, using data from the 2007–2012 waves of the National Health and Nutrition Examination Survey (NHANES).

## 2. Methods

### 2.1. Study Population

The NHANES survey collected cross-sectional data from a representative sample of children and adults from the US population, using a stratified, multistage probability sampling selection approach [18]. Trained interviewers administered questionnaires to participants in their homes on a number of socio-demographic, and healthcare factors. Participants also underwent physical examination, which included clinical measurements (medical, dental and laboratory tests). Detailed descriptions of the methods and protocols utilized in the NHANES surveys are available elsewhere, including informed consent procedures for all participants [19].

For the current analyses, data was utilized in adults (≥18 years of age) who participated in the NHANES 2007–2008, 2009–2010, and 2011–2012 waves. All participants with household FI measures and complete spirometry data that met or exceeded the American Thoracic Society (ATS) data collection standards (FVC = 11,817 or FEV_1_ = 12,175) were eligible for inclusion (n = 12,469).

### 2.2. Outcome Measurements

To measure lung function, participants included in each of the waves between 2007 and 2012 underwent spirometry. The spirometry manouver was performed using the Ohio 822/827 dry-rolling seal volume spirometers. Individuals who reported having chest pain or a physical problems with forceful expiration, were taking supplemental oxygen, had recent surgery, or had recently coughed up blood were excluded. Individuals with a personal history of detached retina or a collapsed lung were also excluded.

Spirometry measurements were graded following the ATS data quality standards. Measures were included in the analyses only if they met or exceeded the ATS standards, which are defined as (A) having three acceptable curves present, and two reproducible curves; and two observed values within 100 mL; or (B) having three acceptable curves present and two reproducible curves; and two observed values within 150 mL) [20].

The outcomes under study were pre-bronchodilator spirometry measures forced expiratory volume in 1 s (FEV_1_), forced vital capacity (FVC), and their ratio (FEV_1_/FVC). Amongst adults aged 40 years of age or older, lower limit of normal (LLN) were calculated to derive spirometrically defined COPD (airflow obstruction, FEV_1_/FVC [<LLN]), and spirometric restriction (FVC < LLN). The calibration equations proposed by Kato et al. to improve validity of pre-bronchodilator measures were used to derive LLN outcomes [21].

### 2.3. Exposures

To ascertain food security at the household level, the NHANES survey used the U.S. Food Security Survey Module questions, self-reported by the adult head of household [22]. The instrument is based on a series of questions related to ability to access food over the previous 12 months, and it included 18 items for households with children or 10 items for households without children. Household food security was categorized based on the number of affirmative responses as full (0), marginal (1–2), low (3–5 for household without children and 3–7 for household with children under the age of 18), and very low (6–10 for household without children and 8-18 for household with children under the age of 18). Similarly, individual food security was categorized into full (0), marginal (1–2), low (3–5) and very low (6–10). For this study, food insecurity categorized into two groups, namely low (full and marginal food security); or high (low, and very low food security).

### 2.4. Other Covariates

The association of FI and lung function outcomes was examined accounting for several potential confounders, including age, sex, height, body mass index (BMI), educational status, annual household income, smoking status, and ethnicity.

BMI was calculated as kg per squared meters (kg/m^2^), and it was categorised as underweight (BMI ≤ 18.4 kg/m^2^); normal (18.5–24.9 kg/m^2^), overweight (25–29.9 kg/m^2^), or obese (≥30 kg/m^2^), as per the Center for Disease Control (CDC) definition. Educational status was categorized as pre-kindergarten, elementary school, middle school, high school, general educational development (GED) or equivalent, and greater than high school. Annual household income was reported as range of value in dollars and re-categorized into four groups: 0–$25 K, $25 K–$55 K, $55 K–$75 K and ≥$75 K. Smoking status was determined using the self-reported smoking status and urinary cotinine levels. Participants were defined as “current”, if had high urinary cotinine (>10 ng/mL), or “former”, if reported individual household smoke exposure or mild urinary levels (≥1 ng/mL and ≤10 ng/mL). Participants were classified as “non-smoker”, if answered “I have never smoked, not even a puff” to the question “Cigarettes smoked in entire life” or who had a urinary cotinine value < 1 ng/mL were classified as never smokers. Ethnicity was classified into two categories: Hispanic (Mexican American and other Hispanic) and non-Hispanic (white, black and other non-Hispanic).

### 2.5. Statistical Analyses

The analyses were adjusted for the sample design, including survey weight, strata and primary sampling design. Continuous and categorical variables were expressed as means ± standard error (SEs) and frequencies, respectively. Mean estimate values of FVC and FEV_1_/FVC ratio was estimated as well as the sample prevalence of COPD (FEV_1_/FVC < LLN), and spirometric restriction (FVC < LLN) for age and sex based on reference equations derived from the third US NHANES [23]. To determine differences by sex, independent T-tests were performed for continuous variables and Chi-squared test for categorical variables. Differences in respiratory outcomes across household FI were assessed in a similar manner.

Population-weighted linear regressions models (β-coefficients) were used to analyze the relationship between household FI and respiratory outcomes. Three models were used: (1) included adjustment for age and height; (2) model 1 + sex; and (3) model 2 + educational status, annual household income, BMI, smoking status and ethnicity. Population-weighted logistic regression models [odds ratios (OR), 95% confidence intervals (95% CI)] were used to investigate the relationship between household FI with COPD (FEV_1_/FVC < LLN) and spirometric restriction (FVC < LLN). Additional analyses stratified by ethnicity (Mexican-American, other Hispanic, non-Hispanic white, non-Hispanic black, and other non-Hispanic) were carried out. Statistical significance was determined as a *p*-value < 0.05. R software was used for all analyses.

## 3. Results

Individuals were on average 43.9 (SE ± 0.3) years of age (Table 1). The prevalence of overweight or obesity was 68.1%, and 29.6% of participants were classified as current smokers. The proportion of household FI was 13.2%.

Statistically significant differences between individuals with high vs. low FI were observed for the mean of FVC (4050 ± 33 vs. 4179 ± 16.5, *p*-value = 0.002), FEV_1_/FVC (0.79 ± 0.004 vs. 0.78 ± 0.002, *p*-value 0.004) and FVC < LLN (3.31 vs. 1.42, *p* < 0.001) (Table 2).

Having high household FI was statistically significantly associated with a lower FVC. This association was observed in baseline model 1 (β = −84.1, 95% CI −124.3, −43.8, *p*-value < 0.001), model 2 (β = −131.2, 95% CI −170.6, −91.7, *p*-value < 0.001) and in the fully adjusted model (β= −70.9, 95% CI −116.6, −25.3, *p*-value = 0.004) (Table 3). High FI was also associated with a lower FEV_1_/FVC ratio (model 1 and model 2, β = −0.008, 95% CI −0.01, −0.002, *p*-value = 0.0125 and *p*-value = 0.0151, respectively), but the statistical significance of these associations were attenuated in the fully adjusted model. Among the LLN outcomes, high household FI was statistically significantly associated with higher odds of spirometric restriction (model 3, OR = 1.02, 95% CI 1.0, 1.03, *p*-value = 0.025), but not with spirometrically defined COPD (Table 3).

The stratified associations by ethnicity between household FI and respiratory outcomes are presented in Supplementary Tables S1–S5. Among Mexican-American, there was a statistically significant negative association between higher household FI and FEV_1_/FVC (model 2, β = −0.005, 95% CI −0.0009, −0.00005, *p*-value 0.036), but the association was attenuated after adjustment for additional confounders (model 3, β = −0.003, 95% CI −0.007, 0.001, *p*-value 0.146). Amongst the ‘Other Hispanics’ group, higher household FI was negatively associated with FVC (model 3, β = −96.5, 95% CI −175.8, −17.2, *p*-value 0.023). Among the ‘Non-Hispanic Blacks’, higher household FI was associated with higher odds of having spirometric restriction (model 3, OR = 1.06, 95% CI 1.01, 1.11, *p*-value = 0.023).

## 4. Discussion

This population-based study in US adults showed that individuals experiencing high FI at the household level had lower lung function, and higher odds of spirometric restriction. The findings also suggest that higher FI was associated with lower FVC and FEV_1_/FVC amongst other Hispanics, and with higher odds of spirometric restriction in non-Hispanic black individuals. These associations remained robust after adjustments for several potential confounders, including age, sex, height, BMI, educational status, annual household income, smoking status, passive/secondhand smoke exposure, and ethnicity.

An accelerated decline in lung function leads to an early onset of COPD. Two other studies have reported an association between FI and COPD. Romero et al. reported a significant association between FI and COPD respiratory symptoms and exacerbations [24]. Specifically, those experiencing greater FI had higher odds of self-reported exacerbations in the previous 3 months (OR = 1.2, *p*-value = 0.02), poorer COPD health status (β = 3.8, *p*-value = 0.01), which was assessed through the COPD Assessment Test [24]. Similarly, using data from the National Health Interview Survey 2011–2015, Gregory et al. showed that adults living in households with greater FI had increased risk of self-reported COPD compared to those in households without FI [25].

Individuals living in households with FI have lower diet quality due to limited accessibility or affordability to acquire healthy foods, including fresh fruits and vegetables. This is often accompanied by increased consumption of high energy dense foods with added sugar, fat, and sodium marketed at more affordable prices [8]. Previous results from the NHANES cycles 1999–2008 indicated that compared to those considered to be food secured, adults with FI consumed more high-fat dairy products, salty snacks, sugar-sweetened beverages, and red and processed meat [26]. On the other hand, FI was associated with lower consumption of vegetables and Healthy Eating Index (HEI)-2005 and HEI-2010 scores, which reflects lower diet quality [26]. In line with these findings, results from the NHANES cycles 2011–2014 showed a negative association between FI and the HEI-2015 score especially in ethnic minority groups [27].

The food environment is an important factor influencing food choices. Food deserts (i.e., a location with inadequate access to healthy foods) and food swamps have emerged among rural towns in the U.S., both of which have been associated with higher consumption of unhealthy diets [28]. The reduction in the consumption of fruits and vegetables can lead to the impairment of lung function and development or progression of COPD. Fruits and vegetables have high amounts of flavonoids and other phenolic compounds with antioxidant and anti-inflammatory properties, which might exert beneficial effects on lung health [15], by reducing the production of pro-inflammatory prostaglandins and leukotrienes [29], which are contributors to impaired lung function [16]. Shaheen et al. found that participants consuming the highest quintile of “prudent” diet score had better lung function in males and females and lower odds of COPD in males [12]. Similarly, results from two large prospective cohort studies, the Health Professionals Follow-up Study in men [14] and the Nurses’ Health Study in women [13] showed that individuals with a prudent dietary pattern had a reduced risk of newly diagnosed COPD. These studies also demonstrated an increased risk of newly diagnosed COPD among participants with the highest quintile of the western pattern compared to those in the lowest quintile group. In European cohorts of adults, the European Community Respiratory Health Survey (ECRHS) found that a higher total intake of fruits was associated with a slower decline in FEV_1_, and a higher tomato intake was associated with a slower decline in FVC [15]. Likewise, authors from the MORGEN study reported that total intake of subclasses of flavonoids catechins, flavonols, and flavones was positively associated with FEV_1_ and negatively associated with breathlessness [16]. Results from the GA^2^LEN survey which included adults from ten European countries, showed that a higher intake of anthocyanins and pro-anthocyanidins was associated with reduced odds of having spirometric restriction (FVC < LLN), whilst compared to those with the lowest intake, individuals with high intake of flavonoids, polymers, and pro-anthocyanidins had better FEV_1_/FVC [17].

FI limits purchasing capability impacting diet quality and may compound the risk of lung function impairment. It has been demonstrated that both FI [27] and COPD manifestation varies according to ethnicity [30]. Accordingly, our results showed significant associations between FI and respiratory outcomes among Other Hispanics, non-Hispanic Blacks, and other non-Hispanics. In US population-based studies, non-Hispanic blacks had the lowest diet quality [27], which might be explained through limited access to healthy food options and that food insecure households had the lowest annual incomes [31], thus compromising the consumption of a healthy diet. It was also found that socioeconomic status, including lower household income was related to a greater prospective risk of COPD exacerbation [32]. Similarly, results from the Genetic Epidemiology of COPD Study demonstrated that African Americans had worse dyspnea and health-related quality-of-life compared to Caucasians and those who experienced exacerbations [30]. In this context, FI might be a risk factor for either unhealthy dietary habits, which in turn can be related to poor lung function or to increase the susceptibility to COPD of some ethnic groups.

The present study has some limitations. The cross-sectional design of the NHANES survey prevents us from establishing causation in the association between FI and lung function outcomes, nor can we evaluate the effect of sustained FI over time on impaired or declining lung function [27]. Considering that household FI was evaluated the previous year, while respiratory outcomes were assessed at the time of the survey, our analyses might be influenced by a misclassification since FI could have changed over the 12 months [26,27].

To our knowledge, this is the first observational study examining the association between FI and lung function outcomes in a nationally representative population of the US. FI was accurately determined based on the USDA Household Food Security Survey Module [26]. Only respiratory measures that exceeded or met the ATS data quality standards for spirometry assessment were included in the analyses. Calibration methods to improve the accuracy and validity of pre- bronchodilator spirometry were utilized when deriving airflow obstruction and spirometric restriction [21]. Objective measures of poor lung function and COPD were evaluated, rather than self-reported or doctor diagnosed COPD, which tends to overestimate and misdiagnose COPD [33].

## 5. Conclusions

These findings suggest that FI is associated with lower lung function, and with a higher odds of having poor respiratory health. Both FI and COPD are major health problems around the world, particularly in settings where health disparities are still a major barrier to wellbeing. As previously suggested, assistance programs to evaluate potential gaps to prevent FI [34] and culturally adapted measures to decrease non-communicable diseases [35] are needed, and should consider the hidden harm caused by FI to respiratory health. Decreasing individual and household FI might contribute to preserving lung function among adults.

## Figures and Tables

**Table 1:** General characteristics of participants.

	Total (n = 12,469)	Males (n = 6224)	Females (n = 6245)	*p*-Value
Age, mean ± SE	43.9 ± 0.4	43.3 ± 0.4	44.5 ± 0.4	<0.001
Height (cm), mean ± SE	169.4 ± 0.1	176.3 ± 0.2	162.7 ± 0.2	<0.001
Body Mass Index (BMI) ^1^ (kg/m^2^), %				<0.001
Underweight	1.6	0.9	2.3	
Normal weight	30.3	26.9	33.6	
Overweight	33.7	39.0	28.5	
Obesity	34.4	33.1	35.6	
Educational Status, %				<0.001
Pre-kindergarten	0	0	0	
Elementary school	0	0	0	
Middle school	0	0	0	
High school	1.1	1.3	0.9	
GED or equivalent	1.2	1.5	1.0	
More than high school	60.8	58.4	63.1	
Missing	36.8	38.8	34,7	
Annual Household Income, %				<0.001
0 to $25,000	17.9	16.1	19.6	
$25,000 to $55,000	26.7	25.8	27.5	
$55,000 to $75,000	13.9	13.0	12.9	
>$75,000	0	0	0	
Missing	42.5	45.0	40.0	
Smoking status ^2^, %				<0.001
Never	48.9	41.8	55.7	
Former	21.6	23.1	20.1	
Current	29.6	35.1	24.2	
Ethnicity, %				<0.001
Hispanic	13.6	14.7	12.6	
Non-Hispanic	86.4	85.6	87.4	
Lung function outcomes				
FVC (mL) ^3^, mean ± SE	4162 ± 14.2	4890 ± 18.8	3468 ± 13.9	<0.001
FEV_1_/FVC (mL) ^4^, mean ± SE	0.78 ± 0.002	0.77 ± 0.003	0.79 ± 0.002	<0.001
FVC < LLN ^5,6^, %	1.61	1.60	1.62	0.934
FEV_1_/FVC < LLN ^6,7^, %	4.99	4.41	5.50	0.149
Household Food Insecurity, %	13.2	13.0	13.4	0.421

^1^ According to the Center for Disease Control (CDC) 2000 growth reference population: underweight (BMI ≤ 18.4 kg/m^2^); normal (18.5–24.9 kg/m^2^), overweight (25–29.9 kg/m^2^), and obese (≥30 kg/m^2^); ^2^ Based on the combination of household smoke exposure and urinary cotinine levels; ^3^ n = 11,817; ^4^ n = 11,523; ^5^ Only among participants aged ≥ 40 years; ^6^ n = 6858, ^7^ n = 6695. GED: General educational development; FVC: Forced Vital Capacity; FEV_1_: Forced Expiratory Volume in the First Second; LLN: lower limit of normal; FVC < LLN: Spirometric restriction; FEV_1_/FVC < LLN: spirometrically defined chronic obstructive pulmonary disease (COPD).

**Table 2:** Differences between respiratory outcomes across food insecurity at household level.

	Household Food Insecurity		
	Low	High	*p*-Value
FVC ^1^, mean ± SE	4179 ± 16.5	4050 ± 33	0.002
FEV_1_/FVC ^2^, mean ± SE	0.78 ± 0.002	0.79 ± 0.004	0.004
FVC < LLN ^3,4^,%	1.42	3.31	<0.001
FEV_1_/FVC < LLN ^3,5^,%	4.80	6.79	0.337

^1^ n = 11,817; ^2^ n = 11,523; ^3^ Only among participants aged ≥ 40 years; ^4^ n = 6858, ^5^ n = 6695. FVC: Forced Vital Capacity; FEV_1_: Forced Expiratory Volume in the First Second; LLN: lower limit of normal; FVC < LLN: Spirometric restriction; FEV_1_/FVC < LLN: Airway obstruction (spirometrically defined chronic obstructive pulmonary disease [CODP]).

**Table 3:** Association between household food insecurity (FI), lung function, COPD and spirometric restriction in adults participating in NHANES.

Respiratory Outcome	Household FI	(95% CI)	*p*-Value
FVC ^1^ (β-coefficient)			
Model 1	−84.1	−124.3; −43.8	<0.001
Model 2	−131.2	−170.6; −91.7	<0.001
Model 3	−70.9	−116.6; −25.3	0.004
FEV_1_/FVC ^2^ (β-coefficient)			
Model 1	−0.008	−0.01; −0.002	0.0125
Model 2	−0.008	−0.01; −0.002	0.0151
Model 3	−0.004	−0.01; 0.002	0.238
FVC < LLN ^3,4,5^ (OR)			
Model 1	1.02	1.01; 1.03	0.002
Model 2	1.02	1.01; 1.04	0.001
Model 3	1.02	1.00; 1.03	0.025
COPD (FEV_1_/FVC < LLN) ^3,6,7^ (OR)			
Model 1	1.03	0.98; 1.07	0.278
Model 2	1.03	0.98;1.08	0.258
Model 3	1.01	0.97; 1.07	0.556

^1^ n = 11,817; ^2^ n = 11,523; ^3^ Only among participants aged ≥ 40 years; ^4^ n = 6858, ^5^ Reference category: FVC ≥ LLN; ^6^ n = 6695; ^7^ Reference category: FEV_1_/FVC ≥ LLN. FVC: Forced Vital Capacity; FEV_1_: Forced Expiratory Volume in the First Second; LLN: lower limit of normal. FVC < LLN: Spirometric restriction; FEV_1_/FVC < LLN: Airway obstruction (spirometrically defined chronic obstructive pulmonary disease [COPD])**.** OR: odds ratio. CI: confidence interval. Model 1: adjusted for age and height; Model 2: additionally adjusted for sex; Model 3: additionally adjusted for educational status, annual household income, body mass index, smoking status, and ethnicity.

## Data Availability

All NHANES data is publicly available.

## Supplementary Materials

The following are available online at https://www.mdpi.com/article/10.3390/nu13062098/s1, Supplementary File 1: Survey Questions Used by USDA to Assess Household Food Security between 2007-2012 from the National Health and Nutrition Examination Survey, Table S1: Association between respiratory outcomes and Food Insecurity (FI) among Mexican American, Table S2: Association between respiratory outcomes and Food Insecurity (FI) among Other Hispanic, Table S3: Association between respiratory outcomes and Food Insecurity (FI) among non-Hispanic white, Table S4: Association between respiratory outcomes and Food Insecurity (FI) among non-Hispanic black, Table S5: Association between respiratory outcomes and Food Insecurity (FI) among other non-Hispanic.
