# Supplementary material for: Household Food Insecurity, Lung Function, and COPD in US Adults
Source: Nutrients. 2021 Jun 19;13(6):2098. doi: 10.3390/nu13062098 (PMC8235683; doi:10.3390/nu13062098)
Supplement: Supplementary file 1 [file nutrients-13-02098-s001.zip › nutrients-1216331-supplementary.pdf]

Supplementary File 1: Survey Questions Used by USDA to Assess Household Food Security between 2007-2012 from the National Health and Nutrition Examination Survey

The following questions were used to assess household food security: 1. "We worried whether our food would run out before we got money to buy more." Was that often, sometimes, or never true for you in the last 12 months?; 2. "The food that we bought just didn't last and we didn't have money to get more." Was that often, sometimes, or never true for you in the last 12 months?; 3. "We couldn't afford to eat balanced meals." Was that often, sometimes, or never true for you in the last 12 months?; 4. In the last 12 months, did you or other adults in the household ever cut the size of your meals or skip meals because there wasn't enough money for food? (Yes/No); 5. (If yes to question 4) How often did this happen—almost every month, some months but not every month, or in only 1 or 2 months?; 6. In the last 12 months, did you ever eat less than you felt you should because there wasn't enough money for food? (Yes/No); 7. In the last 12 months, were you ever hungry, but didn't eat, because there wasn't enough money for food? (Yes/No); 8. In the last 12 months, did you lose weight because there wasn't enough money for food? (Yes/No); 9. In the last 12 months did you or other adults in your household ever not eat for a whole day because there wasn't enough money for food? (Yes/No); and 10. (If yes to question 9) How often did this happen—almost every month, some months but not every month, or in only 1 or 2 months?

The households which included children (<18 years) also considered the following questions: 11. "We relied on only a few kinds of low-cost food to feed our children because we were running out of money to buy food." Was that often, sometimes, or never true for you in the last 12 months?; 12. "We couldn't feed our children a balanced meal, because we couldn't afford that." Was that often, sometimes, or never true for you in the last 12 months?; 13. "The children were not eating enough because we just couldn't afford enough food." Was that often, sometimes, or never true for you in the last 12 months?; 14. In the last 12 months, did you ever cut the size of any of the children's meals because there wasn't enough money for food? (Yes/No); 15. In the last 12 months, were the children ever hungry but you just couldn't afford more food? (Yes/No); 16. In the last 12 months, did any of the children ever skip a meal because there wasn't enough money for food? (Yes/No); 17. (If yes to question 16) How often did this happen—almost every month, some months but not every month, or in only 1 or 2 months?, and 18. In the last 12 months did any of the children ever not eat for a whole day because there wasn't enough money for food? (Yes/No).

References

USDA. Survey Questions Used by USDA to Assess Household Food Security. Available from: http://www.ers.usda.gov/topics/food-nutrition-assistance/food-security-in-the-us/measurement.aspx.

Supplementary Table S1. Association between respiratory outcomes and Food Insecurity (FI) among Mexican American

Respiratory outcome	Household FI (95% CI)	p-value
FVC[1] (β)		
Model 1	62.0 (-0.33; 124.3)	0.058
Model 2	9.69 (-51.7; 71.1)	0.759
Model 3	5.11 (-59.97; 70.18)	0.879
FEV_1/FVC[2] (β)		
Model 1	-0.005 (-0.009; -0.001)	0.021
Model 2	-0.005 (-0.009;-0.0005)	0.036
Model 3	-0.003 (-0.007; 0.001)	0.146

FVC <LLN[3,4,5] (OR)		
Model 1	1.00 (0.9988; 1.0009)	0.327
Model 2	0.9997 (0.9991; 1.0003)	0.316
Model 3	0.9995 (0.0086; 1.0004)	0.301
FEV_1/FVC<LLN[3,6,7] (OR)		
Model 1	1.01 (0.994; 1.02)	0.247
Model 2	1.01 (0.995; 1.02)	0.215
Model 3	1.01 (0.996; 1.02)	0.184

[1]: n=1,952, [2]: n= 1884; [3]: Only among participants aged ≥ 40 years; [4]: n=1026; [5]: Reference category: FVC≥LLN; [6]: n= 992; [7]: Reference category: FEV1/FVC≥LLN

FVC: Forced Vital Capacity;

FEV_1: Forced Expiratory Volume in the First Second

FVC < LLN: Spirometric Restriction

FEV_1/FVC < LLN: Airway obstruction

Model 1: adjusted for age and height

Model 2: additionally adjusted for sex

Model 3: additionally adjusted educational status, annual household income, body mass index and smoking status

Supplementary Table S2. Association between respiratory outcomes and Food Insecurity (FI) among Other Hispanic

Respiratory outcome	Household FI (95% CI)	p-value
FVC[1] (β)		
Model 1	-90.7 (-165; -16.3)	0.022
Model 2	-118.3 (-191.6; -45.0)	0.003
Model 3	-96.5 (-175.8; -17.2)	0.023
FEV_1/FVC[2] (β)		
Model 1	0.006 (-0.003; 0.02)	0.180
Model 2	0.006 (-0.003; 0.02)	0.174
Model 3	0.009 (0.0003; 0.02)	0.051
FVC <LLN[3,4,5] (OR)		
Model 1	1.01 (0.997; 1.023)	0.143
Model 2	1.01 (0.997; 1.023)	0.146
Model 3	1.01 (0.996; 1.024)	0.160
FEV_1/FVC<LLN[3,6,7] (OR)		
Model 1	0.99 (0.97; 1.01)	0.470
Model 2	0.99 (0.97; 1.01)	0.467
Model 3	0.98 (0.96; 1.01)	0.195

[1]: n=1,259; [2]: n= 1227; [3]: Only among participants aged ≥ 40 years; [4]: n=740; [5]: Reference category: FVC≥LLN; [6]: n= 718; [7]: Reference category: FEV1/FVC≥LLN

FVC: Forced Vital Capacity

FEV_1: Forced Expiratory Volume in the First Second

FVC < LLN: Spirometric Restriction

FEV_1/FVC < LLN: Airway obstruction

Model 1: adjusted for age and height

Model 2: additionally adjusted for sex

Model 3: additionally adjusted educational status, annual household income, body mass index and smoking status

Supplementary Table S3. Association between respiratory outcomes and Food Insecurity (FI) among non-Hispanic white

Respiratory outcome	Household FI (95% CI)	p-value
FVC^1 (β)		
Model 1	-77.4 (-129.9; -24.9)	0.006
Model 2	-109.4 (-165.4; -53.4)	<0.001
Model 3	-44.0 (-109.3; 21.4)	0.194
FEV_1/FVC^2 (β)		
Model 1	-0.02 (-0.03; -0.008)	0.002
Model 2	-0.02 (-0.03: -0.007)	0.003
Model 3	-0.008 (-0.02; 0.004)	0.207
FVC <$LLN^{3,4,5}$ (OR)		
Model 1	1.01 (0.997; 1.02)	0.175
Model 2	1.01 (0.997; 1.02)	0.148
Model 3	1.00 (0.989; 1.01)	0.882
FEV_1/FVC<$LLN^{3,6,7}$ (OR)		
Model 1	1.06 (0.98; 1.15)	0.151
Model 2	1.06 (0.98; 1.15)	0.152
Model 3	1.03 (0.95; 1.12)	0.507

[1]: n=5,216; [2]: n= 5111; [3]: Only among participants aged ≥ 40 years; [4]: n=3191; [5]: Reference category: FVC≥LLN; [6]: n= 3132; [7]: Reference category: FEV1/FVC≥LLN

FVC: Forced Vital Capacity

FEV_1: Forced Expiratory Volume in the First Second

FVC < LLN: Spirometric Restriction

FEV_1/FVC < LLN: Airway obstruction

Model 1: adjusted for age and height

Model 2: additionally adjusted for sex

Model 3: additionally adjusted educational status, annual household income, body mass index and smoking status

Supplementary Table S4. Association between respiratory outcomes and Food Insecurity (FI) among non-Hispanic black

Respiratory outcome	Household FI (95% CI)	p-value
FVC[1] (β)		
Model 1	-71.5 (-136.1;-6.89)	0.036
Model 2	-64.9 (-123.4; -6.42)	0.356
Model 3	-58.3 (-120.7; 4.02)	0.075
FEV_1/FVC[2] (β)		
Model 1	-0.007 (-0.01; 0.0006)	0.081
Model 2	-0.006 (-0.01; 0.0006)	0.078
Model 3	-0.0008 (-0.009; 0.007)	0.838
FVC <LLN[3,4,5] (OR)		
Model 1	1.05 (1.01; 1.10)	0.029
Model 2	1.05 (1.01; 1.10)	0.030
Model 3	1.06 (1.01; 1.11)	0.023
FEV_1/FVC<LLN[3,6,7] (OR)		
Model 1	1.00 (0.97; 1.03)	0.894
Model 2	1.00 (0.97; 1.03)	0.893
Model 3	1.00 (0.96; 1.04)	0.990

[1]: n=2,447; [2]: n= 2374; [3]: Only among participants aged ≥ 40 years; [4]: n=1446; [5]: Reference category: FVC≥LLN; [6]: n= 1408; [7]: Reference category: FEV1/FVC≥LLN

FVC: Forced Vital Capacity

FEV_1: Forced Expiratory Volume in the First Second

FVC < LLN: Spirometric Restriction

FEV_1/FVC < LLN: Airway obstruction

Model 1: adjusted for age and height

Model 2: additionally adjusted for sex

Model 3: additionally adjusted educational status, annual household income, body mass index and smoking status

Supplementary Table S5. Association between respiratory outcomes and Food Insecurity (FI) among other non-Hispanic

Respiratory outcome	Household FI (95% CI)	p-value
FVC[1] (β)		
Model 1	26.2 (-100.6; 153.1)	0.687
Model 2	32.2 (-90.9; 155.3)	0.611
Model 3	20.9 (-89.6; 131.44)	0.713
FEV_1/FVC[2] (β)		
Model 1	-0.002 (-0.02; 0.01)	0.761
Model 2	-0.002 (-0.02; 0.01)	0.763
Model 3	0.006 (-0.01; 0.02)	0.362
FVC <LLN[3,4,5] (OR)		
Model 1	0.998 (0.972; 1.025)	0.874
Model 2	0.998 (0.973; 1.025)	0.913
Model 3	1.00 (0.98; 1.022)	0.930
FEV_1/FVC<LLN[3,6,7] (OR)		
Model 1	0.96 (0.94; 0.99)	0.016
Model 2	0.96 (0.94; 0.99)	0.017
Model 3	0.96 (0.92; 0.99)	0.047

[1]: n=943; [2]: n= 927; [3]: Only among participants aged ≥ 40 years; [4]: n=455; [5]: Reference category: FVC≥LLN; [6]: n= 445; [7]: Reference category: FEV1/FVC≥LLN

FVC: Forced Vital Capacity

FEV_1: Forced Expiratory Volume in the First Second

FVC < LLN: Spirometric Restriction

FEV_1/FVC < LLN: Airway obstruction

Model 1: adjusted for age and height

Model 2: additionally adjusted for sex

Model 3: additionally adjusted educational status, annual household income, body mass index and smoking status
